# Case Report of Rare Parathyroid Tumor with Atypical Presentation and Early Diagnosis

**DOI:** 10.1155/2022/1675256

**Published:** 2022-01-11

**Authors:** Érika Mayumi Ikeda Cavamura, Fabiane Karen Miyake, Jéssica Yachio Wiezel, Laura Schwartz Maranho, Luis Felipe Inglês Takada, Renata Cassitas Mendonça, Tamila Sohn Fagundes

**Affiliations:** ^1^Academic of the Faculdade Pequeno Príncipe, Curitiba, Brazil; ^2^Academics of the Faculdade Evangélica Mackenzie Paraná, Curitiba, Brazil; ^3^Medical Residents of the Endocrinology Service at Hospital Universitário Mackenzie Paraná, Curitiba, Brazil

## Abstract

Parathyroid carcinoma is a rare condition, accounting for 1% of cases of hyperparathyroidism. Other causes of hyperparathyroidism main group are single adenoma and parathyroid hyperplasia. The clinics presented by the patients are typical of hyperparathyroidism (fatigue, weakness, weight loss, and anorexia), bone impairment, pain, and fractures, in addition to affecting the renal system The diagnosis of parathyroid carcinoma is most often done postoperatively by means of a histological study. The case report is a 49-year-old male patient who came to the emergency room of Mackenzie Evangelical University Hospital complaining of progressive “muscle weakness” and “joint” that started about 2 months ago. To raise the suspicion of parathyroid carcinoma, it is essential to perform the correlation of the clinical picture, biochemical values, and imaging exams, but to obtain the definitive diagnosis, intraoperative recognition of the tumor and the result of the histopathological examination of the resected tumor are necessary.

## 1. Introduction

Parathyroid carcinoma is a rare condition, accounting for 1% of cases of hyperparathyroidism [1], being described in the literature, less than 600 cases registered up to the year 2001 [2]. Other causes of hyperparathyroidism main group are single adenoma and parathyroid hyperplasia [3].

The patients presented to the clinic are typical of hyperparathyroidism (fatigue, weakness, weight loss, and anorexia), bone impairment, pain, and fractures, in addition to affecting the renal system (nephrolithiasis, nephrocalcinosis, renal colic, and hypercalcemia) [4].

The diagnosis of parathyroid carcinoma is most often done postoperatively by means of a histological study. It can be suspected in the preoperative period through laboratory and clinical findings such as palpable cervical mass (30–76% of cases), vocal fold paralysis, or presence of regional or distance metastasis (lung and liver) [5]. Laboratory tests show hypercalcemia (above 14 mg/dl or 3- 4 mg/dl above normal), parathyroid hormone (PTH) 3–10 times above the normal value, and alkaline phosphatase often increased [4, 6]. The hypothesis of parathyroid carcinoma should be considered in the presence of severe hyperparathyroidism, parathyroidosis, and serum calcium greater than or equal to 14 mg/dl, especially if associated with cervical mass [7]. Fine needle puncture should be avoided in the suspicion of parathyroid carcinoma due to the risk of metastatic local [8].

The importance of suspecting preoperative carcinoma is mainly due to its management being different from adenoma [9]. Major differences between carcinoma and other benign parathyroid diseases include mean age (48 and 55 years, respectively), tumor size >3 cm frequent in carcinoma and rare in benign diseases, very high PTH in carcinoma and slightly elevated in disease benign, palpable cervical mass present in carcinoma, and rare in benign diseases [10].

The treatment is surgical, with extensive resection with tumor block whenever malignant neoplasia is suspected. It should include the parathyroid, the ipsilateral thyroid lobe, the isthmus, and the lymph nodes of the central compartment. Cervical emptying should be performed in the presence of metastasis. In the postoperative period, it is important to control hipocalcemia [5]. Metabolic complications such as hypercalcemic crisis, bone, or renal abnormalities are the most common [11].

## 2. Case Report

A 49-year-old male came to the emergency room of Mackenzie Evangelical University Hospital complaining of progressive “muscle weakness” and “joint” that started about 2 months ago. The pain occurs in the lumbar spine region associated with nausea, diarrhea (6 episodes), hyporexia, fluctuating fecal acholia, and unintentional weight loss of about 20 kilograms (kg) within 3 months. He denies allergies, smoking, drinking, and other comorbidities. Physical examination showed arthralgia in the lumbar spine without pain on palpation, but painful with flexion and extension of the spine. He also had pain on palpation of the right lower thoracic region and right hypochondrium and a slightly enlarged and slow base gait with a palpable thyroid nodule in the left lobe region. He refers to an uncle with a history of bone carcinoma. Patient was admitted to the intensive care unit (ICU) with optimized analgesia, hydration, and furosemide.

**Figure 1:**
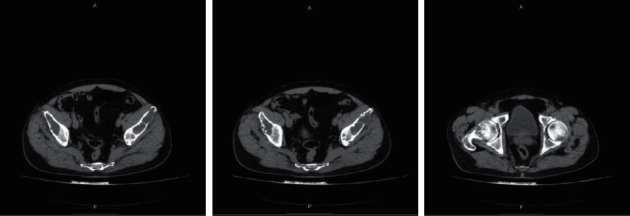
Chest CT scans.

**Figure 2:**
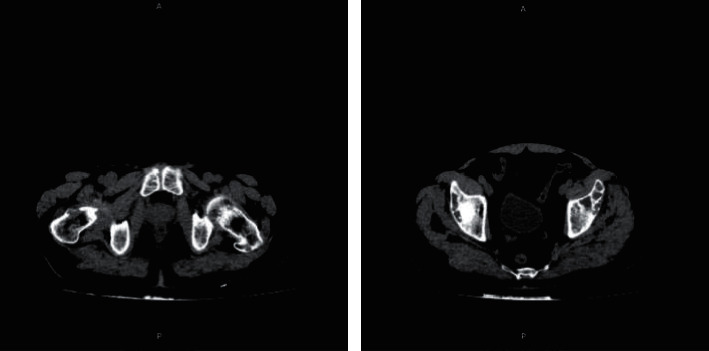
Contrast abdomen CT scans.

**Figure 3:**
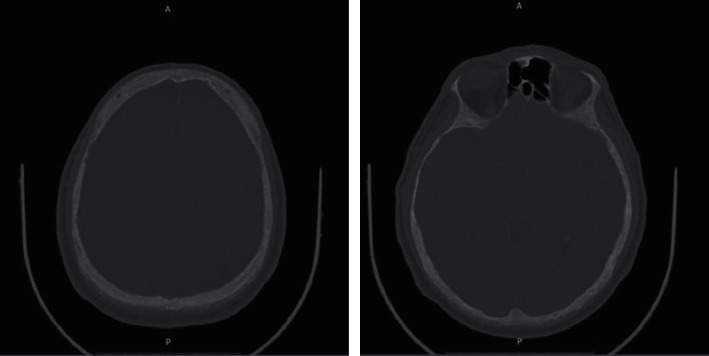
Cranial CT scans.

Chest (1), contrast abdomen (2), and skull (3) computed tomography (CT) and CPK scans were ordered, which showed the following, respectively. (Figures 1–3)

1. Lytic lesion in costa arch and right trochanter, sprouting tree-like lung tissue with calcified granulomas, and absence of pulmonary lymph nodes or nodules
2. Diffuse lytic lesions in the bilateral pelvis and right fender head, associated with no obstructive bilateral nephrolithiasis
3. Reduction of diffuse bone density of skull cap without lytic lesions

Serious calcium was also measured with results greater than 17.1 mg/dl, PTH of 2627 pg/ml, and 558 alkaline phosphatase and hypophosphatemia, as well as protein electrophoresis, creatine phosphokinase test (CPK), prostate-specific antigen (PSA), and partial urine.

Due to hypercalcemia, pamidronate was prescribed with relative improvement of calcium level investigation followed with USG of the cervical region demonstrating a mass in the cervical region on the left, quite delimited, heterogeneous, predominantly hypoechoic, with macrocalcifications and beam fibrotics in between, presenting exuberant flow to the study doppler with apparent plan of cleavage and thyroid measuring 3.0 × 3.5 × 3.0 cm, without being possible to remove the possibility of injury of nature parathyroid among the hypotheses.

Endocrinology follow-up indicates daily dosages of calcium (Ca), phosphorus (P), magnesium (Mg), albumin, and correction as needed, vitamin D dosage, and electrocardiogram (EKG), indication for postoperative ICU parathyroidectomy due to the risk of severe hypocalcemia (“bone hunger”), preoperative examinations, and dosage intraoperating of PTH, besides immediate beginning of calcitriol (dose attacks 2 mcg/day) and cholecalciferol 50.000 UI for 3 consecutive days before the surgery and to maintain maintenance dose with 10.000 UI in a week and magnesium and furosemide replacement.

A parathyroidectomy of the lower left lobe associated with level VI lymphadenectomy was performed. The lesion was highly adhered posteriorly to the sternal part of the sternocleidomastoid muscle and was extremely solid. The level of PTH and vitamin D in the preoperative one was of 2657 pg/ml and 24 ng/ml and in the immediate postoperative thing of 325 pg/ml and 36 ng/mL. Calcium gluconate, EV, and bicarbonate were maintained, as well as vitamin D2 and calcium carbonate replacement with neurological surveillance (epilepsy/tetany/paresthesias), cardioscopy, and prevention of hyperventilation/alkalosis with guidance not to perform loop diuretics and prophylactic cefazolin. The material sent to anatomopathological showed epithelial neoplasia, sometimes trabecular, and sometimes nodular with rounded nucleus cells and macronucleolus, presenting capsule infiltration, without invasion of adjacent tissue or necrosis, with 3 mitoses in 50 GCA.

In immunohistochemistry, it is neoplasia with focal perivascular neoplastic extension that corresponds to vascular invasion and observes capsular transposition and fibrous bands in a trabecular pattern amidst neoplasia. These parameters may correlate with more aggressive clinical behavior, including the occurrence of metastases. The possibility of parathyroid carcinoma should be clinically positive and researched with appropriate evolutionary, clinical, and laboratory monitoring, despite presenting histological aspects that can be correlated with atypical parathyroid adenoma and presenting the following immunohistochemical panel. (Table 1)

Calcitriol 0.25 mcg 3 cp/day associated with cholecalciferol 10,000 IU/week was maintained, and calcium IV replacements were performed; serum calcium, phosphate, and magnesium measurements were performed for follow-up, which showed progressive improvement. The patient was referred to the room after 3 days of ICU with a suspension of calcium IV replacement and started calcium citrate associated with oral vitamin D3. At the seventh postoperative day, PTH was 26 pg/ml and continued improvement in other laboratory tests. New skull tomography demonstrated calcification in the right frontal lobe with signs of bone thinning in skull cap. (Table 2)

**Figure 4:**
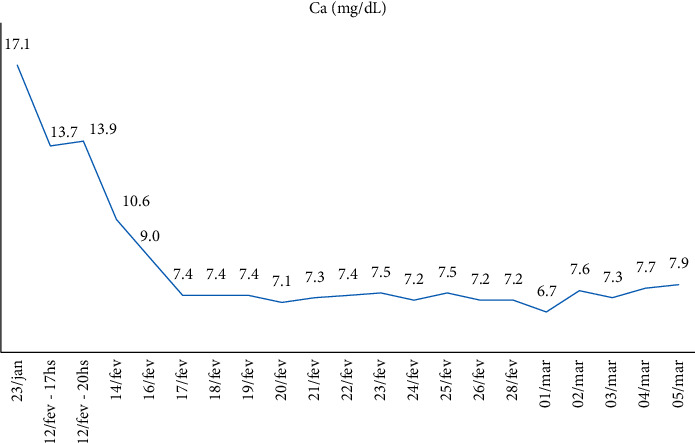
Evolution of the patient's calcium level.

**Figure 5:**
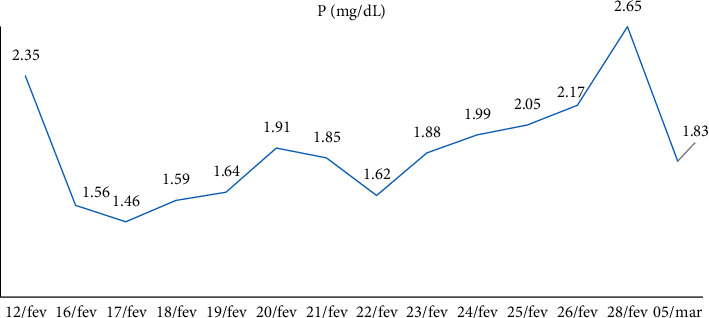
Evolution of patient's phosphate level.

**Figure 6:**
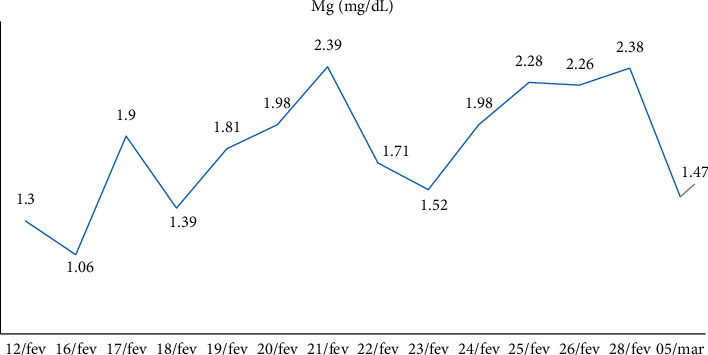
Evolution of patient's magnesium level.

Hospital discharged the patient after 42 days of hospitalization. The patient was referred for follow-up at the endocrinology outpatient clinic, where he presented weight gain and muscle strength, but maintaining the need for a walking stick and with asymptomatic hypocalcemia. (Figures 4–6)

## 3. Discussion

The parathyroid gland consists of a set of glands that are located mainly in the apex and in the inferior poles of the right and left lobes of the thyroid, usually in a total of four [12]. They originate from the third and fourth branchial arches in the embryonic period. The involvement of this gland by neoplasia is defined as parathyroid carcinoma, an endocrine tumor that has unknown etiology and is associated with mutations in the HRPT2/CDC73 gene [13]. The present case proved to be a parathyroid carcinoma located in the inferior left lobe of the thyroid, requiring parathyroidectomy associated with lymphadenectomy level VI.

The involvement of this carcinoma is not predisposed by sex [14]. The age range of patients at diagnosis varies between 28 and 72 years, with a higher frequency at 40–50 years [8]. The patient in the reported case was male and 49 years old, that is, within the most frequent range of the tumor. The evolution of the neoplasm is slow and progressive with a high recurrence rate. The prognosis depends on the success of the surgical procedure and early detection. The case demonstrates carcinoma discovered at earlier stages in which the moment of follow-up at the endocrinology outpatient clinic showed no recurrence. According to data from the National Cancer Database, the estimated 5 year survival is 49% [15].

In the physical examination of most cases, as well as in the reported case, the tumor is a palpable—a rare differential in cases of benign adenoma hyperparathyroidism. Laboratory tests show elevated calcium levels greater than 14.0 mg/dL or an increase of 3-4 mg/dL in normal index, parathyroid hormone levels of 500 ng/dL or an increase of 3–10 times higher than normal values, and hypophosphatemia [10]. The reported tumor presented levels around 17 mg/dL at the time of diagnosis and a significant increase in parathyroid hormone levels with a preoperative 2657 pg/ml, in addition to hypophosphatemia.

When a diagnosis of parathyroid carcinoma is suspected, at least two imaging tests are recommended to obtain the precise location of the tumor. The most common techniques are cervical ultrasound and Sestamibi, 99m Tc scintigraphy [9, 16]. Ultrasound examination is used to locate the disease, analyze local recurrence, and as a guide for fine needle aspiration (FNA) when regional lymph node metastasis is suspected. However, the FNAB technique should be performed with caution or avoided, since there is a risk of tumor spread in the biopsy path [9].

Already, the scintigraphy with Sestamibi, 99m Tc can indicate the place that the disease affects, however, though it is not possible to distinguish carcinoma from a benign disease. Computed tomography (CT) and magnetic resonance imaging (MRI) may indicate metastases and recurrence cases [17].

Radiography shows bone changes in the hands in 83% of the patients, in the skull in 71%, and in the spine and long bones in 90% of them [18]. Radiological changes are consistent with fibrous osteitis and subperiosteal resorption. The imaging exams in this case were chest, abdomen, and skull scans, all showing radiological alterations. From the anatomopathological point of view, they present macroscopically as large tumors, with diameters greater than 1.5 cm, adherent to adjacent tissues and frequently presenting invasion of the ipsilateral thyroid lobe and cervical musculature [14]. On microscopy, they present thick acellular fibrous bands, mitotic activity, vascular invasion, and capsular invasion [19]. The anatomopathological observation involves the difficulty of differentiating carcinoma from parathyroid adenoma, as in both situations, nuclear pleomorphism, hyperchromatism, free tumor cells in blood vessels, mitosis, and giant cells can be found [20]. Thus, achieving an accurate diagnosis is difficult due to the diversity of clinical presentations and examination findings [21]. The material sent to the anatomopathological presented fields of trabecular and nodular epithelial neoplasia with round nucleus cells and macronucleolus, presenting capsule infiltration, without adjacent tissue invasion or necrosis. This shows that despite the capsule infiltration, the diagnosis was early, since it did not present adjacent tissue invasion.

To raise the suspicion of parathyroid carcinoma, it is essential to perform the correlation of the clinical picture, biochemical values, and imaging exams, but to obtain the definitive diagnosis, intraoperative recognition of the tumor and the result of the histopathological examination of the resected tumor are necessary [22].

The intraoperative macroscopic characteristics of the tumor can be evaluated. Thus, in benign tumors such as parathyroid, adenomas have characteristics such as oval shape, elastic consistency, and reddish color. Already, carcinoma tends to present as a firm mass with gray-white fibrous capsule adhering to the thyroid lobe and other cervical tissues—laryngeal nerve, infrahyoid muscles, esophagus, and trachea [17, 22].

However, the absence of local invasion or regional metastasis makes differentiation difficult [17]. In addition, in freezing analysis, the histopathological aspects of carcinoma may overlap with those of parathyroid adenoma. Therefore, when there is a high preoperative suspicion for carcinoma and absence of suspicious intraoperative findings, en bloc resection of the tumor is recommended [17, 22].

It is not uncommon for a patient with parathyroid carcinoma to be diagnosed only when local recurrence or distant metastasis occurs [22]. Thus, in cases of severe hyperparathyroidism, carcinoma should always be investigated to make an early diagnosis when there is still a possibility of cure.

Adjuvant irradiation of the neck and mediastinum has recently been reported to be useful in reducing the risk of locoregional disease progression and improving survival [23, 24] as well as for palliative symptoms of hypercalcemia in inoperable patients [2]. In this case, the patient was considered healed and is on watch for possible relapse.

When there is no possibility of surgical correction, clinical treatment can be performed to reduce hypercalcemia and minimize bone resorption. The therapy is aimed at inhibiting bone release of calcium and increasing urinary excretion, and the three targets for treatment are the tumor, osteoclast, and kidney. Initial treatment includes vigorous saline hydration. Loop diuretics, calcitonin, bisphosphonates, plicamycin or mitramycin, and calciomimetic agents [2] may be used. In this case, hydration, loop, diuretic, calcitriol, cholecalciferol, magnesium, calcium, parathyroidectomy, and serial serum calcium, phosphate, and magnesium dosages were performed until discharge.

Metastases occur late, with lymphatic and hematogenous spread, and may occur in the lung, liver, bones, and pancreas [11]. Local invasion to contiguous structures is more frequently observed [18]. Death usually results from metabolic complications associated with hypercalcemia [14], which in this case was of great concern and follow-up by the hospital service.

Follow-up aimed at early detection of locoregional recurrence and/or distant metastases [25, 26]. It should be done by monitoring calcium and PTH every 2 years for the first 5 years and then annually associated with annual cervical ultrasound [25].

The 5 and 10-year survival rate is around 77–100% and 66–80%, respectively [27]. However, the local recurrence rate is common, from 40% to 63% [27]. Many may have had recurrence due to inadequate surgical resection or due to nondiagnosis of the tumor preoperatively [27].

Lymph node involvement or distant metastasis are associated with a higher recurrence rate [27]. Most patients have local recurrence, and many of these require multiple surgeries with a high risk of complications, especially in relation to the recurrent laryngeal nerve [27, 28].

Silva-Figueroa and others created a score for prognostic assessment in patients with parathyroid carcinoma [29]. Age greater than 65 years, serum calcium >15 mg/dl, and vascular invasion were negatively correlated with relapse-free survival after initial parathyroidectomy [29]. Further validation of this score is still needed in larger cohort studies to assess its clinical applicability [29]. Patient W.G.'s only poor prognostic factor was serious calcium, which was around 17 mg/dl.

The cause of mortality is usually due to complications of intractable hypercalcemia [25, 27–29], which leads to renal failure, arrhythmias, or pancreatitis [28].

Up to 25% of patients with parathyroid carcinoma may develop distant metastases during follow-up [27]. The most affected sites are the lung, bones, and liver [26, 27].

The prognosis is variable, but solid tumors seem to have better survival than nonsolid tumors [27].

The prognosis of nonfunctioning parathyroid carcinoma is uncertain due to its rarity, but it seems to have a worse prognosis, since they are diagnosed later in more advanced staging [25, 27]. The presence of lymph node metastases is generally associated with a worse prognosis, and therefore, appropriate cervical dissection should be performed in these patients [27]. The patient in the reported case had an early diagnosis in which he was associated with lymphadenectomy.

Other prognostic factors include age, gender, time to first relapse, elevated serum calcium on recurrence, number of relapses, number of medications used to decrease calcium, inability to completely resect the tumor, and tumor aneuploidy [27, 29].

## 4. Conclusion

Parathyroid carcinoma is a rare malignant tumor that tends to have clinical signs of severe hypercalcemia. Because it is an uncommon cancer, it is difficult to access consistent data on morbidity and mortality.

The diagnosis is difficult, being necessary the suspicion of the disease preoperatively or even during surgery to enable the most effective treatment, and the definitive diagnosis is not rare only when there is metastasis or recurrence, which highlights the follow-up of these patients. Surgical treatment at an early stage of the disease is an important factor for disease control. Complete block resection with eventual hemithyroidectomy and ipsilateral centrocervical lymphadenectomy should represent the minimal oncologic approach in all patients with suspected parathyroid carcinoma, significantly improving disease-free survival. However, relapse is often not the cause of poor course, and the major cause uncontrollable hypercalcemia is the most common cause of death. In addition, recommendations or protocols for the management of parathyroid carcinoma are not yet available, and further multicenter studies are essential to improve the knowledge and treatment of this cancer.

## Figures and Tables

**Table 1:** Immunohistochemical panel.

AE1/AE3	Positive
Calcitonin	Negative
CD31	Positive in vessel
Chromogranin A	Negative
ERG	Positive in vessel
GATA3	Positive
Ki-67	Positive in 10% of cells
Paratormon	Positive
PAX-8	Positive focal
Synaptophysin	Positive focal
Thyroglobulin	Negative
TTF-1	Negative

**Table 2:** Evolution of the patient's level of albumin, PTH, creatinine, and vitamin D in the preoperative and postoperative periods.

12/02-17H	ALB: 3.7	
12/02-20H	ALB: 3.3, CR: 1.48	
14/02	PTH: 325	
17/02	CR: 1.16	
18/02	CR: 1.20	
PTH	Pre-op: 2657	Immediate postoperative period: 325 7oPO: 26
Vitamin D	Pre-op: 24	Post-op: 36
